# A review of epidemiological parameters from Ebola outbreaks to inform early public health decision-making

**DOI:** 10.1038/sdata.2015.19

**Published:** 2015-05-26

**Authors:** Maria D. Van Kerkhove, Ana I. Bento, Harriet L. Mills, Neil M. Ferguson, Christl A. Donnelly

**Affiliations:** 1 MRC Centre for Outbreak Analysis and Modelling, Department of Infectious Disease Epidemiology, Imperial College London, London W2 1PG, UK; 2 Center for Global Health, Institut Pasteur, Paris 75015, France

**Keywords:** Epidemiology, Ebola virus, Viral epidemiology, Viral infection

## Abstract

The unprecedented scale of the Ebola outbreak in West Africa has, as of 29 April 2015, resulted in more than 10,884 deaths among 26,277 cases. Prior to the ongoing outbreak, Ebola virus disease (EVD) caused relatively small outbreaks (maximum outbreak size 425 in Gulu, Uganda) in isolated populations in central Africa. Here, we have compiled a comprehensive database of estimates of epidemiological parameters based on data from past outbreaks, including the incubation period distribution, case fatality rate, basic reproduction number (*R*_*0*_), effective reproduction number (*R*_*t*_) and delay distributions. We have compared these to parameter estimates from the ongoing outbreak in West Africa. The ongoing outbreak, because of its size, provides a unique opportunity to better understand transmission patterns of EVD. We have not performed a meta-analysis of the data, but rather summarize the estimates by virus from comprehensive investigations of EVD and Marburg outbreaks over the past 40 years. These estimates can be used to parameterize transmission models to improve understanding of initial spread of EVD outbreaks and to inform surveillance and control guidelines.

## Background & Summary

Ebola virus disease (EVD), formerly known as Ebola hemorrhagic fever, is caused by a zoonotic virus first discovered in 1976 in remote villages of Democratic Republic of Congo (DRC, formerly Zaire) and Sudan^1–4^. The virus was again identified in the mid-1990s, when it re-emerged in Gabon and Kikwit, DRC^5,6^. Since then, there have been sporadic outbreaks in human and non-human-primate populations, primarily in remote areas in central Africa (Table 1)^7–9^. There are five Ebola viruses: *Zaire ebolavirus*, *Sudan ebolavirus*, *Bundibugyo ebolavirus, Tai Forest ebolavirus and Reston ebolavirus*. The most lethal is the Ebola Zaire virus. Ebola Reston is unique among the five Ebola viruses in that it is not known to cause disease in humans^10^ and *Ebola Tai Forest* has only been reported in 1 human case^11^. Together with Marburg virus, Ebola forms the Filoviridae family (filovirus)^8^.

The primary reservoir of the Ebola virus is believed to be fruit bats^12,13^. However, non-human primates, including chimpanzees, gorillas, and cynomolgus monkeys, and forest antelopes have been reported as possible vectors in transmission to humans^14^, and EVD has caused devastating mortality in non-human-primate populations^15^. Once infected, the symptoms of human EVD are non-specific and typically include fever, headache, joint or muscle pain, sore throat, vomiting, and/or diarrhea^15–17^. More severe cases involve hemorrhagic manifestations, shock and other neurological symptoms^14,16–21^.

While it has been difficult to trace the source of human outbreaks, it is believed that EVD outbreaks usually start from a zoonotic source with subsequent human-to-human transmission^22,23^. Transmission between humans occurs through exposure to infectious bodily fluids, typically from close contact with infectious individuals when caring for EVD patients (e.g., sharing of contaminated needles, family home care, insufficient protective measures among health care workers in health care settings^6,24,25^) or with fatal EVD patients in preparation for burial^19,20^. Control measures for EVD are well documented and include identification, isolation and care of suspected patients, strict infection prevention and control among those caring for patients and safe burials^26,27^.

At the start of an infectious disease outbreak, it is critical to understand the transmission dynamics of the pathogen and to determine those at highest risk for infection or severe outcomes in the population(s) affected^28,29^. This information is needed to develop interventions to reduce the spread of disease and to reduce morbidity and mortality in the affected populations. Real-time analysis of any ongoing outbreak by analyzing detailed information collected on the confirmed, probable and suspected cases and deaths provides an opportunity to determine the stages of disease and areas where control measures can be applied. For example, knowledge of the incubation period distribution of the pathogen will inform the duration of time required to follow up the contacts of cases to evaluate whether or not they become secondary cases. Additionally, information on the timing of symptom onset, isolation, hospitalization and outcome (either death or recovery) are important to understand EVD progression. Mathematical models which make use of available data early in an outbreak to estimate the outbreak’s potential impact are increasingly used by public health policy makers to inform decision making around emerging and re-emerging pathogens^28–30^.

The purpose of this review was to collect all published epidemiological parameter estimates (reprinted in detailed tables containing estimates, and corresponding confidence intervals) estimated from past EVD outbreaks. Our aim was not to perform a meta-analysis, but rather to compile and document the available parameter estimates based on data from EVD outbreaks over the past 40 years. In order to estimate any of the parameters referenced in our manuscript, we would need detailed case data of each of the cohorts studied in the original papers, which we do not have. We also reprint parameter estimates from past Marburg outbreaks and the ongoing outbreak in West Africa for comparison. This information is valuable for public health organizations that need to quickly evaluate the early behavior of a new outbreak and estimate the potential impact, in terms of morbidity, mortality and geographic spread. We highlight how the parameter estimates we have examined improve our understanding of EVD epidemiology. Our results help to put the ongoing EVD outbreak in West Africa into context and to assess the likely effects of ongoing and novel interventions.

## Methods

### Data collection

All searches using the following search terms (Ebola, Marburg, EHF, EVD, MHF, EBOV, *Ebola Zaire*, *Ebola Sudan*, *Ebola Reston*, *Ebola Bundibugyo*, outbreak, model, parameterization, incubation period, case fatality rate, case fatality rate (CFR), risk factors, basic reproduction number, *R*_*0*_, effective reproduction number, serial interval, delay distributions, generation time) were carried out on 1 August 2014, 15 September 2014 and again in February 2015 using the following databases: ScienceDirect, ResearchGate, Google, GoogleScholar, BioOne, Web of Science and PubMed. Our searches aimed to find primary reports describing and analyzing data collected from investigations of EVD and Marburg outbreaks since the virus was identified in 1976. The criteria for inclusion were: sample size of EVD cases described in the study ≥5, studies of human outbreaks, studies which evaluated potential risk factors had to report prevalence proportion ratios, odds ratios or relative risks. Reviews, commentaries, case reports on individual cases, and policy pieces were excluded. Additionally, literature evaluating non-human outbreaks or the potential for international (human) spread of EVD outside of an outbreak zone was excluded.

Using these search terms, a total of 49 papers were determined eligible for inclusion. In addition, for context we included additional published information on EVD including the final outbreak sizes as reported by the World Health Organization (WHO) Disease Outbreak News following declaration that each outbreak was over.

From the relevant EVD and Marburg literature, we extracted the following details for all parameter estimates (as provided): point estimates, confidence intervals, ranges, sample size used to estimate the parameter (total numbers of cases encompassing confirmed, suspected, and retrospectively diagnosed cases, depending on the study), EVD virus, and inferential methods. We then compiled the parameter estimate database into tables. Table 1 and Data Citation 1 list the human outbreaks of *Ebola Zaire*, *Ebola Sudan* and *Ebola Bundibugyo* that have occurred in Africa from 1976 to present. We have not provided detailed information on the outbreaks as these have been previously described^9^. Table 2 (available online only) summarizes the literature we used in this review.

Our manuscript and tables include estimates, confidence intervals and ranges obtained from the referenced publications (Table 2 (available online only) and Data Citation 2).

### Definition of key parameters recorded

The incubation period is the interval between exposure to a pathogen and initial occurrence of symptoms and signs^28,29^. The incubation period distribution is usually characterized using the mean or the median incubation period.

The CFR is the proportion of cases (infected symptomatic individuals) within a designated population who die as a result of their infection. For past EVD and Marburg outbreaks, we report on the CFR estimated after the outbreak was declared over (estimated at least 42 days after the last case experienced symptom onset) by taking the number of deaths among cases divided by the total number of cases recorded during the outbreak. However, during outbreaks, the CFR is often estimated before all cases have been identified and before some cases have either recovered or died.

Risk factors for infection include demographic factors, medical conditions and behavioral exposures or practices that are associated with an individual’s risk of becoming infected with Ebola.

The basic reproduction number (*R*_*0*_) is used to measure the transmission potential of a disease. It is the average number of secondary infections produced by an infected case in a susceptible population^31^. If *R*_*0*_ >1, then once established the outbreak will continue, whereas if *R*_*0*_<1, then the outbreak will die out.

The effective reproduction number (*R*_*t*_) is similar to *R*_*0*_ but relates to a particular calendar time *t* (after the start of the outbreak). Like *R*_*0*_, if *R*_*t*_>1, then the outbreak will continue, whereas if *R*_*t*_<1, then the outbreak will die out. *R*_*t*_ can be reduced through the use of successful control measures (e.g., by limiting contacts between susceptible and infectious individuals). *R*_*t*_ can also be reduced due to the depletion of susceptible individuals whether through extensive transmission or through the immunization of susceptible individuals^32^.

The serial interval is the interval between symptom onset in an index case and symptom onset in a secondary case infected by that index case^33^.

The generation time is the interval between infection of an index case and infection of a secondary case infected by that index case. The serial interval is more frequently estimated than the generation time and is often assumed to be the same duration as the generation time^34^.

### Delay distributions

1. *Symptom onset to hospitalization (also referred to as onset to clinical assessment):* The interval between symptom onset and hospitalization.
2. *Hospital admission to day of first blood sample:* The interval between admission to hospital or medical facility for treatment of EVD and when a biological sample is collected for diagnosis.
3. *Symptom onset to recovery/discharge:* The interval between symptom onset and recovery or hospital discharge.
4. *Symptom onset to death:* The interval between symptom onset and death.
5. *Duration of admission (survivors)—hospitalization to discharge:* The interval between admission to a hospital or medical facility for treatment of EVD and discharge from the facility.
6. *Duration of admission (fatal cases)—hospitalization to death:* The interval between admission to a hospital or medical facility for treatment of EVD and death.

## Data Records

The data from this analysis are summarized in two types of data format. Four data tables detail the methods and parameter estimates from each study included in our review. Our data tables:

1. Table S1: *Human Outbreaks of Ebola Zaire, Ebola Sudan and Ebola Bundibugyo from 1976* presents compiled data on the year and location of the each human outbreak, the Ebola Virus causing the outbreak and number of cases reported (Data Citation 1).
2. Table S2: *Parameter Estimates by Outbreak* presents a comprehensive list of parameter estimates, including incubation period distribution, reproduction number, serial interval distribution, generation time distribution, delay distributions, and CFR by Ebola virus and study (Data Citation 2).
3. Table S3: *Parameter estimates for the ongoing EVD outbreak in West Africa* presents published estimates of delay distributions and CFR for the ongoing outbreak in West Africa (Data Citation 3).
4. Table S4: *Risk factors for EVD and Marburg infection* presents published results from outbreak (Data Citation 4).

**Figure 1:**
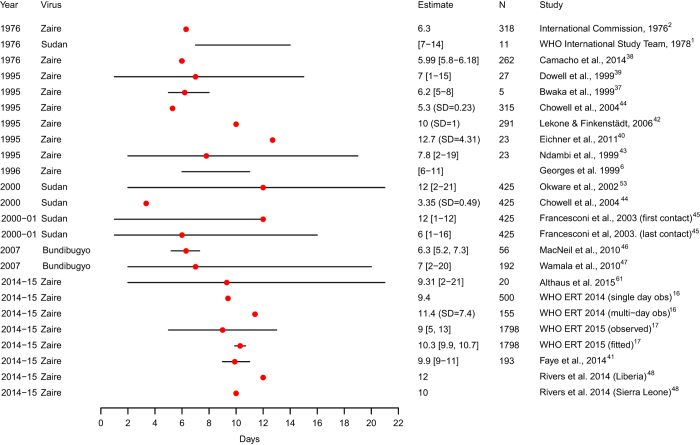
Estimates of the incubation period distribution by virus, year of outbreak and study. The dots represent the mean or median estimate and the lines illustrate the range, for all studies, with the exception of MacNeil *et al.* ^46^ and WHO Ebola Response Team (ERT) 2015^17^ (fitted) where the line represents the 95% CI for the estimate. Chowell *et al.* ^44^, Eichner *et al.* ^40^, WHO ERT 2014 (multi-day observed)^16^ and Lekone and Finkenstädt^42^ provide standard deviation (s.d.).

**Figure 2:**
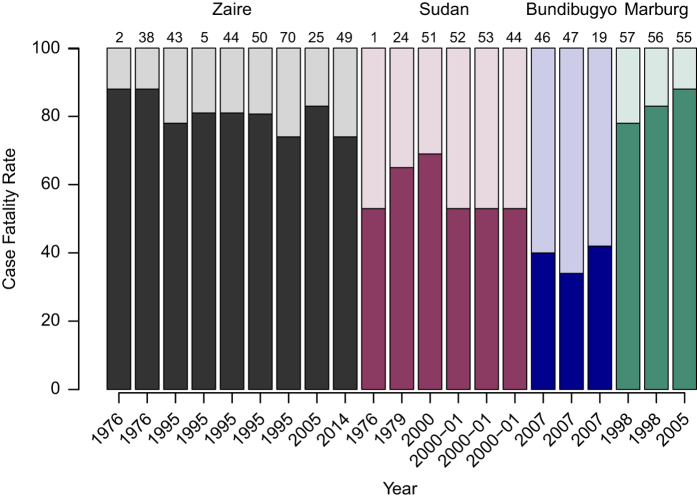
Overall case fatality rate (CFR) for Ebola by virus and Marburg virus; we include only those outbreaks that have been declared over. Outbreaks with fewer than 10 fatal patients were excluded from this figure. For each outbreak, the bars represent the rate of fatal (dark color) and recovered (light color) patients. For country specific information see Table 1. The source of each estimate is denoted by the reference number at the top of each bar.

Using these four tables, we then summarized the parameter database in six tables and two figures presented in this article. The parameters estimated for *Ebola Zaire*, *Ebola Sudan* and *Ebola Bundibugyo* outbreaks, including the incubation period distribution, serial interval distribution, *R*_*0*_, delay distributions and CFR, are shown in Tables 3 (available online only), 4 (available online only), 5, respectively. Parameter estimates for the ongoing outbreak in West Africa are summarized in Table 6 (available online only) and for Marburg outbreaks are presented in a single table (Table 7). Risk factors for Ebola and Marburg infection are summarized in Table 8 (available online only). Estimates of the incubation period distribution and CFR are presented in Figs 1 and 2, respectively.

## Technical Validation

### Incubation period distribution

The incubation period distribution of EVD has been estimated for past EVD outbreaks (Fig. 1 and Tables 3 (available online only), 4 (available online only), 5; minimum sample size *n*=5, maximum sample size *n*=1,798). The mean (or median) incubation period (Fig. 1), for the different Ebola viruses ranged from 3.35 to 12.7 days (range 1–21 days), excluding an extreme outlier^35^. Central estimates for the incubation period distribution were between 5.3–12.7 days (range 1–21 days) for *Ebola Zaire* ^5,6,16,17,36–43^, 3.35–12 days (range 1–16 days) for *Ebola Sudan* ^1,44,45^, and 6.3–7 days (range 2–20 days) for *Ebola Bundibugyo* ^46,47^.

The mean incubation period for the ongoing Ebola outbreak in West Africa has been estimated to be between 9–12 days (Table 6 (available online only))^16,17,41,48^. The range of incubation periods observed in past EVD outbreaks supports the policy of contact tracing for 21 days following contact with an EVD patient. An outbreak is officially declared over after no new cases are identified 42 days (2 times the 21-day maximum incubation period) after the last EVD case is found.

### Case fatality rate (CFR)

In Fig. 2 and Tables 3 (available online only), 4 (available online only), 5, we reprint the estimated CFR for each Ebola outbreak (by virus) and for Marburg virus. The *Ebola Zaire* virus is the most lethal with an overall estimated CFR ranging from 69 to 88%^2,5,25,38,43,49,50^ (Table 3 (available online only)). The CFR of outbreaks due to *Ebola Sudan* virus ranged from 53 to 69%^1,24,51–53^ (Table 4 (available online only)), and the CFR of outbreaks due to *Ebola Bundibugyo* ranged from 34 to 42%^19,46,47^ (Table 5). For the ongoing outbreak in West Africa due to *Ebola Zaire*, the estimated CFR, as measured among confirmed and probable cases with definitive outcome (recovered or fatal), is approximately 70%, and varies little among the three most affected countries (Guinea, Liberia and Sierra Leone; Table 6 (available online only) and Data Citation 2)^38^. The CFR among EVD cases reported by Nigeria (*n*=20) was 40%^54^). A second, unrelated EVD outbreak occurred in Équateur province, DRC between July and October 2014 resulting in 69 confirmed and probable cases with a CFR of 74%^49^. The CFR for Marburg is approximately 80%^55–57^).

### *R*_*0*_ and *R*_*t*_

Looking at past outbreaks, estimates of *R*_*0*_ for *Ebola Zaire* ranged from 1.4-4.7^35,42,44,58–60^) (Table 3 (available online only) and Data Citation 2), and for *Ebola Sudan* ranged from 1.3-2.7^44,58–60^ (Table 4 (available online only)). *R*_*0*_ has not been estimated for *Ebola Bundibugyo*.

In the ongoing outbreak in West Africa, estimates of *R*_*0*_ and *R*_*t*_ have been estimated for all countries combined, as well as separately for Guinea, Liberia, Nigeria and Sierra Leone^16,30,36,41,48,54,61–69^. All estimates of *R*_*t*_ are provided in Table 6 (available online only) and Data Citation 2 and Data Citation 3. Gomes *et al.* ^62^ reported several all-country *R*_*0*_ estimates (means ranging 1.8–2.1), depending on model choice and assumptions. Fisman *et al.* ^30^ reported all-country and country-specific *R*_*0*_ estimates depending on assumptions including action taken to mitigate infection. For the most part, *R*_*0*_ estimates for Guinea, Liberia, and Sierra Leone ranged from 1.2 to 2.5 with the striking exception of the Fisman *et al.* ^30^ *R*_*0*_ estimate of 8.3 for Sierra Leone. Nishiura and Chowell^65^ estimated *R*_*t*_ fluctuating around 1 for Guinea, 1.7 for Liberia and 1.4 for Sierra Leone. The WHO Ebola Response Team^16^ estimated *R*_*t*_ for Guinea (ranging from 1.6 to 2.0), for Liberia (ranging from 1.4 to 1.6) and for Sierra Leone (ranging from 1.3 to 1.5).

Several groups have also estimated *R*_*0*_ for specific geographic areas within the region (full details in Table 6 (available online only) and Data Citation 2). For example, Faye *et al.* estimated *R*_*0*_ for cases occurring in Conakry, Guinea at the start of the outbreak (*n*=193)^41^ as 1.7 (95% CI 1.2, 2.3); whereas Lewnard *et al.* ^64^ estimated *R*_*0*_ for EVD cases in Montserrado, Liberia as of October 2014 (*R*_*0*_=2.5 (2.4, 2.6)).

### Serial interval distribution

The serial interval, defined as the time interval between symptom onset in an index case and symptom onset in a secondary case infected by that index case, has been infrequently estimated due to the paucity of data on epidemiologically linked pairs of index and secondary cases. For *Ebola Zaire* (Table 3 (available online only)), the mean serial interval was estimated to be 10–16.1 days^5,49,60,70^. In the ongoing outbreak in West Africa, the mean serial interval has been estimated to be approximately 14–15 days^16,17,30,41^ (Table 6 (available online only)).

### Generation time distribution

Closely related to the serial interval, the generation time is defined as the time interval between infection of an index case and infection of a secondary case infected by that index case. As such, the generation time distribution nearly always needs to be inferred indirectly from serial interval observations and knowledge of the incubation period distribution. We found one such estimate of the mean generation time for Marburg of 9 days (95% CI 8.2, 10.0)^55^.

### Delay distributions

For *Ebola Zaire*, including the ongoing outbreak, the mean time from symptom onset to hospitalization (Table 3 (available online only) and Table 6 (available online only)), ranged from 3.2 to 5.3 days^5,16,17,20,38,41,48^, whereas the mean time from symptom onset to death ranged from 6 to 10.1 days^5,17,25,37–39,41,49,61^. For *Ebola Sudan* (Table 4 (available online only)), the mean time from symptom onset to hospitalization was 2 days (range 0–8)^51^ and the median time from symptom onset to death was 9 days (range 5–15)^24^, respectively. The mean time from hospitalization to discharge for *Ebola Sudan* ranged from 8 to 10 days^51,53^ whereas the mean time from hospitalization to death was 6.1 days (range 2–13)^51^. For *Ebola Bundibugyo* (Table 5), the median time from symptom onset to hospitalization was 3.5 days (range 0–8)^19^ and the median time from symptom onset to death was 9–10 days (range 3–21 days)^19,47^.

### Risk for developing EVD

Risk factors for human-to-human transmission of EVD or Marburg were evaluated from comparison of the exposures, behaviors and practices in cases compared to controls (including unaffected controls, defined to be suspected cases but negative serologic test results) and were described using a prevalence proportion ratio, an odds ratio or a relative risk (and the corresponding confidence interval). Significant risk factors associated with developing EVD are reported in Table 8 (available online only) and Data Citation 4 and include direct physical contact (sharing a bed, touching a cadaver or funeral preparations for an EVD patient, nursing care and contact with bodily fluids) and non-physical contact (sharing a meal, contact with a hospital where EVD patients were treated)^24,39,45,47,56,71^.

## Usage Notes

The data presented in this review summarize estimates of the epidemiological parameters of EVD and Marburg. These results can facilitate parameterization and sensitivity analysis of transmission models examining surveillance, control and treatment strategies. The results can also inform epidemiological studies investigating human-to-human transmission during Ebola and Marburg outbreaks, deepening our understanding of the transmission process.

The number of parameters estimated for each outbreak has generally increased with time (Table 2 (available online only)). While the incubation period distribution was consistently assessed, *R*_*0*_ has increasingly been estimated, most notably with the ongoing outbreak in West Africa (Table 6 (available online only) and Data Citation 2). Fig. 1 shows the central estimates and ranges for the different studies that estimated the incubation period distribution for EVD outbreaks. While there are small differences in the central estimates of the incubation period distribution of the four Ebola viruses, the ranges around the mean or median are consistent, with a maximum of ≤21 days. Current EVD guidance states that EVD has an incubation period of 2–21 days, which is the basis for the recommended duration of contact tracing of 21 days^26,27^. This is supported by the findings in our review.

Figure 2 shows CFR for different *Ebola Zaire*, *Ebola Sudan, Ebola Bundibugyo* and Marburg outbreaks. While the CFR for Ebola and Marburg are high (compared to other infectious diseases), outbreaks caused by *Ebola Zaire* and *Ebola Sudan* have experienced the highest CFR amongst these three Ebola viruses causing outbreaks in humans^1,2,5,19,24,25,38,43,46,47,50–53,72–76^. The estimated CFR for the ongoing outbreak in West Africa, due to *Ebola Zaire*, is approximately 70%^16,17,36^, which falls within the range of CFRs for past outbreaks due to this virus^2,5,25,38,43,50^.

Figure 2 also illustrates that recent CFR estimates for *Ebola Zaire* remain comparable to those observed in the 1970s. While there are ongoing efforts to develop medical treatments for EVD, treatment remains mainly supportive. The massive scale of the ongoing outbreak has highlighted the urgent need to develop new treatments and to fast track the use of experimental medical interventions^77^.

The transmission potential, as measured by *R*_*0*_, is fairly consistent among the three Ebola viruses, ranging from approximately >1 to 4 (also mentioned in ref. 78). Previously, EVD typically affected villages in remote areas of central Africa^35,38,42,44,58,59^, and while devastating in these areas, the populations that are at risk are generally limited in number. The ongoing EVD outbreak had circulated for at least three months prior to discovery^22,79^ which allowed spread of the virus to go unchecked while it infected people in an area of Guinea that shares borders with Sierra Leone and Liberia. Recent experience in Nigeria, has shown that an Ebola virus with *R*_*0*_>1, even in a population of over 20 million, can be controlled with vigorous application of control methods^49,54^.

Differences in estimates of *R*_*0*_ and *R*_*t*_ are likely, at least in part, to be the result of the quality of data available and the inferential method. The focus on *R*_*0*_ estimation together with serial interval estimates may reflect a shift from data collection purely for surveillance to recognition of the epidemiological value of such data.

The specific factors that result in an EVD outbreak have been under investigation since the emergence of this virus and include examination of human and susceptible non-human populations living in close proximity with each other in remote areas of central Africa. Recent investigations into the first cases of the ongoing outbreak found that the outbreak may have begun in Meliandou, Guinea in a village where the inhabitants frequently came in contact with fruit bats in a hollowed out tree^80^. Although the current focus is on limiting human-to-human transmission and treating the infected, the challenging underlying factors that led to this large outbreak in West Africa will require long-term investments to improve both health care and surveillance for infectious diseases.

Our dataset is the most complete collection of published epidemiological parameter estimates from EVD outbreaks available at the time of writing and provides an evidence-based foundation for both retrospective analyses and responses to future outbreaks.

## Additional Information

**How to cite this article:** Van Kerkhove, M. D. *et al.* A review of epidemiological parameters from Ebola outbreaks to inform early public health decision-making. *Sci. Data* 2:150019 doi: 10.1038/sdata.2015.19 (2015).

## Supplementary Material



## Figures and Tables

**Table 1:** Human Outbreaks of *Ebola Zaire*, *Ebola Sudan* and *Ebola Bundibugyo* from 1976 to present

**Outbreak**	**Year**	**Ebola virus**	**Number of confirmed, probable and suspected cases**
Yambuku, DRC^2^	1976	Zaire	318
South Sudan^1^	1976	Sudan	284
Nzara, Sudan^24^	1979	Sudan	34
Gabon^6^	1994–1995	Zaire	49
Kikwit, DRC^5^	1995	Zaire	315
Maybout, Gabon^*^	1996	Zaire	37
Booue, Gabon^6^	1996–1997	Zaire	60
South Africa^74^	1996	Zaire	2
Gulu, Uganda^45,52,53^	2000–2001	Sudan	425
Republic of Congo and Gabon^75^	2001–2002	Zaire	65 in Gabon, 59 in Congo
Republic of Congo^25,72^	2002–2003	Zaire	143
Mbomo, Republic of Congo^25^	2003	Zaire	35
Yambio, South Sudan^†^	2004	Sudan	17
Etoumbi, Republic of Congo^25^	2005	Zaire	12
Kasai Occidental Province, DRC^23^	2007	Zaire	264
Bundibugyo District, Uganda^47^	2007–2008	Bundibugyo	116
Kasai Occidental Province, DRC^‡^	2008–2009	Zaire	32
Orientale Province, DRC^§^	2012	Bundibugyo	77
Kibaale District, Uganda^||^	2012	Sudan	24
Luwero District, Uganda^¶^	2012–2013	Sudan	7
Équateur province, DRC^49^	2014	Zaire	69
West Africa^#^	2014–2015	Zaire	>26,000
Outbreaks with more than 1 case are included in the table.			
DRC=Democratic Republic of Congo; The outbreak in West Africa includes cases from the following countries: Guinea, Liberia, Mali, Nigeria, Senegal and Sierra Leone, Spain, United Kingdom, and United States.			

*Reference: WHO 26 April 1996 Disease Outbreak News. 1996- Ebola haemorrhagic fever in Gabon- Update3: www.who.int/csr/don/1996_04_26b/en/.

^†^Reference: WHO 7 August 2004: WHO announces end of Ebola Outbreak in Southern Sudan: www.who.int/csr/don/2004_08_07/en/.

^‡^Reference: WHO Disease Outbreak News: www.who.int/csr/don/2009_02_17/en/.

^§^Reference: www.cdc.gov/vhf/ebola/outbreaks/history/summaries.html.

^||^Reference: WHO Disease Outbreak News: www.who.int/csr/don/2012_09_03/en/.

^¶^Reference: WHO Disease Outbreak News: www.who.int/csr/don/2012_11_30_ebola/en/.

^#^Outbreak is ongoing in Guinea, Liberia and Sierra Leone. Case count reflects cases reported as of 29 April 2015.

**Table 2:** List of studies used in the review and the estimated parameters

			**Estimated Parameters**							
**Study**	**Virus**	**Year ofoutbreak**	**Incubation PeriodDistribution**	**R_0_**	**R_t_**	Serial Interval^*^Distribution	**GenerationTime Distribution**	**Delay Distributions**	**CFR**	**RFI**
International Commission^2^	*EBOV Zaire*	1976	x						x	x
Camacho *et al.* ^38^	*EBOV Zaire*	1976	x	x				x	x	
WHO International Study Team^1^	*EBOV Sudan*	1976	x						x	
Baron *et al.* ^24^	*EBOV Sudan*	1979						x	x	x
Dowell *et al.* ^39^	*EBOV Zaire*	1995	x			x		x		x
Bwaka *et al.* ^37^	*EBOV Zaire*	1995	x					x		
Chowell *et al.* ^44^	*EBOV Zaire and Sudan*	1995; 2000–2001	x	x				x	x	
Lekone & Finkenstädt^42^	*EBOV Zaire*	1995	x	x						
Eichner *et al.* ^40^	*EBOV Zaire*	1995	x							
Ndambi *et al.* ^43^	*EBOV Zaire*	1995	x						x	
Khan *et al.* ^5^	*EBOV Zaire*	1995				x		x	x	
Muyembe & Kipasa^70^	*EBOV Zaire*	1995				x			x	
Legrand *et al.* ^59^	*EBOV Zaire and Sudan*	1995; 2000–2001		x						
Ferrari *et al.* ^58^	*EBOV Zaire*	1995		x						
Sadek *et al.* ^50^	*EBOV Zaire*	1995						x	x	x
Rowe *et al.* ^20^	*EBOV Zaire*	1995						x		
Bertherat *et al.* ^71^	*EBOV Zaire*	1995								x
Ndanguza *et al.* ^35^	*EBOV Zaire*	1995	x	x						
White & Pagano^60^	*EBOV Zaire*	1995		x		x				
Georges *et al.* ^6^	*EBOV Zaire*	1996	x					x		
Okware *et al.* ^53^	*EBOV Sudan*	2000–2001	x					x	x	x
Borchert *et al.* ^51^	*EBOV Sudan*	2000						x	x	
Lamunu *et al.* ^52^	*EBOV Sudan*	2000–2001							x	x
Francesconi *et al.* ^45^	*EBOV Sudan*	2000–2001	x							x
Nkoghe *et al.* ^25^	*EBOV Zaire*	2005						x	x	
MacNeil *et al.* ^46^	*EBOV Bundibugyo*	2007	x						x	
Wamala *et al.* ^47^	*EBOV Bundibugyo*	2007	x					x	x	x
Roddy *et al.* ^19^	*EBOV Bundibugyo*	2007						x	x	
Althaus^36^	*EBOV Zaire*	2014–2015		x					x	
Gomes *et al.* ^62^	*EBOV Zaire*	2014–2015		x						
Fisman *et al.* ^30^	*EBOV Zaire*	2014–2015		x		x				
Nishiura & Chowell^65^	*EBOV Zaire*	2014–2015			x					
WHO Ebola Response Team^16^	*EBOV Zaire*	2014–2015	x	x	x	x		x	x	
WHO Ebola Response Team^17^	*EBOV Zaire*	2014–2015	x		x	x		x	x	
Althaus *et al.* ^61^	*EBOV Zaire*	2014–2015	x	x				x	x	
Fasina *et al.* ^54^	*EBOV Zaire*	2014–2015		x	x				x	
Faye *et al.* ^41^	*EBOV Zaire*	2014–2015	x	x		x		x	x	
Lewnard *et al.* ^64^	*EBOV Zaire*	2014–2015		x						
Towers *et al.* ^66^	*EBOV Zaire*	2014–2015			x					
Maganga *et al.* ^49^	*EBOV Zaire*	2014			x	x		x	x	
Webb *et al.* ^68^	*EBOV Zaire*	2014–2015		x						
White *et al.* ^69^	*EBOV Zaire*	2014–2015			x					
Yamin *et al.* ^76^	*EBOV Zaire*	2014–2015			x					
Rivers *et al.* ^48^	*EBOV Zaire*	2014–2015	x	x				x	x	
Colebunders *et al.* ^57^	*Marburg*	1998						x	x	
Bausch *et al.* ^56^	*Marburg*	1998							x	
Ajelli & Merler^55^	*Marburg*	2005		x			x	x	x	
CFR refers to case fatality rate.										
RFI refers to risk factor for infection.										

*Some of the studies refer to this parameter estimate as generation time but actually estimate serial interval.

**Table 3:** Parameter Estimates for *Ebola Zaire* excluding the ongoing outbreak in West Africa

**Parameter**	**Estimate**
**Incubation Period Distribution (range of central estimates, (range))** ^*^	5.3–12.7 (1–21) days
**Serial interval Distribution (range of mean estimates)**	10–16.1 days
*Khan et al. (mean)* ^†^^5^	14 days^‡^
*Muyembe & Kipasa (approximation)* ^†^^70^	10 days‡
*Dowell et al. (median, (range))* ^†^^39^	Med=17 days (9–25)
*White & Pagano (mean, (IQR))* ^60^	5.82 days (5.43–7.60)
*Maganga et al. (median, (range), mean, s.d.)* ^49^	Med=16 days (3–27), 16.1 days, 4.4
***R***_***0***_ **(range of estimates)**	1.36–4.71
*Chowell et al. (estimate, s.d.)* ^44^	1.83, 0.06
*Ferrari et al. (estimate, (95% CI)* ^58^	3.65 (3.05, 4.33)
*Legrand et al. (estimate, (95% CI))* ^59^	2.7 (1.9, 2.8)
*Lekone & Finkenstädt (estimate, s.d.)* ^42^	1.36, 0.13
*Ndanguza et al. (estimate, (95% CI))* ^35^	2.22 (1.90, 2.73)
*White & Pagano (estimate, (IQR))* ^60^	1.93 (1.74–2.78)
*Camacho et al. (estimate, (95% CI))* ^38^	4.71 (3.92, 5.66)
***R***_***t***_ **(range of estimates)**	0.84–1.29
*Maganga et al. (estimate, (95% CI)* ^49^)	1.29 (−4.72, 7.29)0.84 (−0.38, 2.06)
**Delay Distributions (days)**	
Infectious period	
*Chowell et al. (mean, s.d.)* ^44^	5.61,0.19
Symptom onset to…	
… hospitalization (range of mean and median estimates)	4–5; Med=3–4
*Khan et al. (mean, median, (range), n)* ^5^	5, Med=4 (0–19), *n*=219
*Rowe et al. (mean,* s.d.*, median, (range))* ^20^	4, 3.3, Med=3 (0–14)
*Camacho et al. (median, (95% CI))* ^38^	Med=3.00 (2.81, 3.20)
… death (range of mean estimates)	6–10.1
*Camacho et al. (median, (95% CI))* ^38^	Med=7.49 (7.30, 7.69)
*Bwaka et al. (mean, (range), n)* ^37^	10.1 (3–21), *n*=86
*Dowell et al. (median)* ^39^	Med=10
*Khan et al. (mean, median, (range), n)* ^5^	9.6, Med=9 (0–34), *n*=224
*Nkoghe et al. (mean, (range), n)* ^25^	6.2 (3–13), *n*=12
	Med=6 (<15 years old)
	Med=9 (15–29 years old)
*Sadek et al. (median, n)* ^50^	Med=10 (30–44 years old)
	Med=8 (45–59 years old)
	Med=9.5 (>59 years old), overall *n*=226
*Georges et al. (range)* ^6^	12–18
*Maganga et al. (median, (range), mean, s.d.)* ^49^	11 (1–30), 11.3, 6.8
*… recovery*	10
*Camacho et al. (median, (95% CI))* ^38^	10.00 (9.80, 10.19)
Hospitalization to…	
… discharge	17
*Khan et al. (mean, median, (range), n)* ^5^	17, Med=14 (0–56), *n*=34
… death	4.6
*Khan et al. (mean, median, (range), n)* ^5^	4.6, Med=4 (0–20), *n*=185
Death to burial	
*Camacho et al. (median, (95% CI))* ^38^	Med=0.99 (0.8, 1.18)
**Overall Case Fatality Rate (range of estimates)^α^**	0.69–0.88
*International Commission (estimate, n, year of outbreak)* ^2^	0.88, *n*=318, 1976
*Muyembe & Kipasa (estimate, n, year of outbreak)* ^70^	0.74, *n*=136, 1995
*Ndambi et al. (estimate, n, year of outbreak)* ^43^	0.78, *n*=23, 1995
*Khan et al. (estimate, n, year of outbreak)* ^5^	0.81, *n*=315, 1995
	Overall: 0.807, *n*=310, 1995
	0.778 (<15 years old)
*Sadek et al. (estimate, n, year of outbreak)* ^50^	0.69 (15–29 years old)
	0.796 (30–44 years old)
	0.89 (45–59 years old)
	0.957 (>59 years old)
*Chowell et al. (estimate, n, year of outbreak)* ^44^	0.81, *n*=315, 1995
*Nkoghe et al. (estimate, n, year of outbreak)* ^25^	0.83, *n*=12, 2005
*Camacho et al. (estimate, n, year of outbreak)* ^38^	0.88, *n*=262, 1976
*Maganga et al. (estimate, n, year of outbreak)* ^49^	0.74, *n*=69, 2014
Med=median; s.d.=standard deviation.	

*See Fig. 1 for individual estimates; extreme outlier estimate from Ndanguza *et al.* 35 not included.

^†^generation time estimated as serial interval.

^‡^No methodology provided.

α*See Fig. 2 for CFR estimates.

**Table 4:** Parameter Estimates for *Ebola Sudan*

**Parameter**	**Estimate**
**Incubation Period Distribution (range of central estimates, (range))** *	3.35–14 (2–21) days
***R***_***0***_ ***(range of estimates)***	1.34–2.7
*Chowell et al. (estimate,* s.d.)^44^	1.34, 0.03
*Legrand et al. (estimate, (95% CI))* ^59^	2.7 (2.5, 4.1)
*Ferrari et al. (estimate, (95% CI))* ^58^	1.79 (1.52, 2.30)
**Delay Distributions (days)**	
Infectious period…	
*Chowell et al. (mean, s.d.)* ^44^	3.50,0.67
Symptom onset to…	
… hospitalization	2
*Borchert et al. (mean, (range), n)* ^51^	2 (0–8), *n*=26
… death	9
*Baron et al. (median, (range), n)* ^24^	Med=9 (5–15), *n*=22
… discharge	12
*Okware et al. (mean, (range), median)* ^53^	12, (2–35), 13
Hospitalization to…	
… discharge (range of mean estimates)	8–10
*Borchert et al. (mean, (range), n)* ^51^	8.0 (2–11) *n*=8
*Okware et al. (mean, (range))* ^53^	10 (1–29)
… death	6.1
*Borchert et al. (mean, (range), n)* ^51^	6.1 (2–13) *n*=18
**Case Fatality Rate** ^†^ **(range of estimates)**	0.53–0.69
*WHO International Study Team (estimate, n, year of outbreak)* ^1^	0.53, *n*=284, 1976
*Baron et al. (estimate, n, year of outbreak)* ^24^	0.65, *n*=34, 1979
*Okware et al. (estimate, n, year of outbreak)* ^53^	0.53, *n*=425, 2000–2001
*Lamunu et al. (estimate, n, year of outbreak)* ^52^	0.53, *n*=425, 2000–2001
*Chowell et al. (estimate, n, year of outbreak)* ^44^	0.53, *n*=425, 2000–2001
*Borchert et al. (estimate, n, year of outbreak)* ^51^	0.69, *n*=26, 2000
Med=Median; s.d.=standard deviation.	

*See Fig. 1 for individual estimates.

^†^See Fig. 2 for CFR estimates.

**Table 5:** Parameter Estimates for *Ebola Bundibugyo*

**Parameter Estimate**	**Estimate**
**Incubation Period Distribution (days) (mean, (95% CI), median, (range))** *	6.3 (5.2, 7.3), Med=7 (2–20)
**Delay Distributions (days)**	
Symptom onset to…	
… hospitalization	3.5
*Roddy et al. (median, (range), n)* ^19^	Med=3.5 (0–8), *n*=26
… death (range of medians)	9–10
*Roddy et al. (median, (range), n)* ^19^	Med=9 (3–20), *n*=11
*Wamala et al. (median, (range), n)* ^47^	Med=10 (3–21), *n*=(NP)
…recovery	10
*Wamala et al. (median, (range), n)* ^47^	Med=10 (2–26), *n*=(NP)
**Case Fatality Rate** ^†^ **(range of estimates)**	0.34–0.42
*MacNeil et al. (estimate, n, year of outbreak)* ^46^	0.40, *n*=43^‡^, 2007
*Wamala et al. (estimate, n, year of outbreak)* ^47^	0.34, *n*=116, 2007
*Roddy et al. (estimate, n, year of outbreak)* ^19^	0.42, *n*=26 (hospitalized confirmed), 2007–08
Med=median; (NP)=Not Provided.	

*See Fig. 1 for individual estimates.

^†^See Fig. 2 for CFR estimates.

^‡^Confirmed cases only.

**Table 6:** Parameter estimates for the ongoing outbreak in West Africa

**Parameter**	**All countries** *	**Guinea**	**Liberia**	**Nigeria**	**Sierra Leone**	**References**
**Incubation Period Distribution (days)**						
Multi-day exposures, observed (mean)	11.4, *n*=155	10.9, *n*=20	11.7, *n*=79	—	10.8, *n*=48	^16^
Single-day exposures, observed, (median (IQR))	9 (5–13)					^17^
Fitted single-day exposures (mean, (95% CI))	10.3 (9.9, 10.7)					^17^
Fitted values (mean)			12		10	^48^
**Serial interval Distribution (days)**						
*WHO ERT 2014 (gamma fit mean, s.d., n)*	15.3, 9.3, *n*=92)	19.0, 11.2, *n*=40)	13.1, 7.8, *n*=26)	—	11.6, 6.3, *n*=25)	^16^
*WHO ERT 2015 (gamma fit mean, (95% CI), n)*	14.2 (13.1, 15.3), *n*=305					^17^
*Fisman et al.* ^30^	15 days (derived)					^30^
*Faye et al. 2014 (mean, (range), s.d., (95% CI for s.d.))*		14.2 (13.1–15.5), 7.1 (6.2, 8.2)				^41^
***R*_*0*_ (estimate, (95% CI))**						
With estimated serial interval 15.3 d	—	1.71 (1.44, 2.01)	1.83 (1.72, 1.94)		2.02 (1.79, 2.26)	^16^
With fixed incubation period 5.3 d	—	1.51 (1.50, 1.52)	1.59 (1.57, 1.60)	9.0 (5.2, 15.6)†	2.53 (2.41, 2.67)	^36,61^
Overall, data driven	1.8 (1.5, 2.0)	—	—	—	—	^62^
SEIR model	2.1 (1.9, 2.4)	—	—	—	—	^62^
Community using Legrand *et al.* ^59^ model	0.8 (0.3, 0.9)	—	—	—	—	^62^
Hospital using Legrand *et al.* ^59^ model	0.4 (0.2, 1.4)	—	—	—	—	^62^
Funeral using Legrand *et al.* ^59^ model	0.6 (0.2, 1.0)	—	—	—	—	^62^
Assumed fixed generation time of 15d	1.78	2.46	1.72	—	8.33	^30^
Estimated mean serial interval 15.3d	—	1.71 (1.44, 2.01)	1.83 (1.72, 1.94)	1.2 (0.67, 1.96)	2.02 (1.79, 2.26)	^16^
Overall (Conakry)		1.7 (1.2, 2.3)				^41^
Community (Conakry)		1.0 (0.6, 1.5)				^41^
Hospital (Conakry)		0.4 (0.2, 0.7)				^41^
Funeral (Conakry)		0.3 (0.1, 0.6)				^41^
Montserrado only			2.49 (2.38, 2.60)			^64^
*Webb et al. 2015*			1.54		1.26	^68^
*Rivers et al.* ^48^ *(overall)*			2.22		1.78	^48^
*Rivers et al.* ^48^ *(community)*			1,35		1.11	^48^
*Rivers et al.* ^48^ *(hospitals)*			0.35		0.24	^48^
*Rivers et al.* ^48^ *(funerals)*			0.53		0.43	^48^
*Khan et al.* ^63^ *(raw data)*			1.76		1.49	^63^
*Khan et al.* ^63^ *(corrected for underreporting)*			1.9		1.37	^63^
***R*_*t*_ (estimate, (95% CI))**						
Estimated mean serial interval 15.3 d	—	1.81 (1.60, 2.03)	1.51 (1.41, 1.60)	<1†	1.38 (1.27, 1.51)	^16,36^
Estimated mean generation time 12 d^‡^	—	1	1.7	—	1.4	^65^
Sierra Leone (56 days of data)					1.1 (0.95, 1.24)	^69^
Montserrado only			1.73 (1.66, 1.83)			^76^
Survivors Montserrado only			0.66 (0.10, 1.69)			^76^
Non-survivors Montserrado only			2.36 (1.72, 2.80)			^76^
**Observed Delay Distributions (days)**						
**Symptom onset to…**						
**… hospitalization**						
*WHO ERT 2014 (mean, s.d., n)*	5.0, 4.7, *n*=1135	5.3, 4.3, *n*=484	4.9, 5.1, *n*=245	4.1, 1.4, *n*=11	4.6, 5.1, *n*=395	^16^
*WHO ERT 2015 (mean, (95% CI), n)*	5.0 (4.9, 5.1), *n*=5616					^17^
*Faye et al. 2014 (mean, s.d., n)*		5.0, 3.9, *n*=152				
*Rivers et al.* ^48^ *(mean)*			3.24		4.12	^48^
**... hospital discharge**						
*WHO ERT 2014 (mean, s.d., n)*	16.4, 6.5, *n*=267	16.3, 6.1, *n*=152	15.4, 8.2, *n*=41	—	17.2, 6.2, *n*=70	^16^
*WHO ERT 2015 (mean, (95% CI))*	15.1 (14.6, 15.6)					^17^
**… death**						
*WHO ERT 2014 (mean, s.d., n)*	7.5, 6.8, *n*=594	6.4, 5.3, *n*=248	7.9, 8, *n*=212	—	8.6, 6.9, *n*=128	^16^
*WHO ERT 2015 (mean, (95% CI))*	8.2 (7.9, 8.4)					^17^
*Faye et al. 2014 (mean, s.d., n)*		8.9, 4.0, *n*=82)				^41^
*Althaus et al.* ^61^ *(mean, range)*				7.41 (4–17), *n*=17		^61^
**… WHO notification**	6.1, 8.5, *n*=2185	7.5, 10.4, *n*=743	6, 8.7, *n*=797	—	4.5, 5, *n*=634	^16^
**WHO notification to…**						
**… hospital discharge** *(mean, s.d., n)*	11.8, 7.2, *n*=312	11.1, 5.8, *n*=164	11, 8, *n*=41	—	12.7, 8.4, *n*=102	^16^
**… death** *(mean, s.d., n)*	-3.0, 13.8, *n*=584	-4.4, 14.4, *n*=300	-1.8, 13.6, *n*=221	—	-1.6, 9.2, *n*=58	^16^
**Hospitalization to…**						
**… discharge**						
*WHO ERT 2014 (mean, s.d., n)*	11.8, 6.1, *n*=290	11, 5.4, *n*=159	12.8, 8.1, *n*=40	—	12.4, 5.8, *n*=86	^16^
*WHO ERT 2015 (mean, (95% CI))*	11.2 (10.8, 11.7)					^17^
*Rivers et al.* ^48^ *(recovery) (mean)*			15.88		15.88	^48^
**… death**						
*WHO ERT 2014 (mean, s.d., n)*	4.2, 6.4, *n*=121	2.5, 3.4, *n*=36	4.5, 6, *n*=63	—	4.4, 6, *n*=17	^16^
*WHO ERT 2015 (mean, (95% CI))*	4.3 (4.1, 4.5)					^17^
*Rivers et al.* ^48^ *(mean)*			10.07		6.26	^48^
**Infectious period**						
*Rivers et al.* ^48^ *(mean)*			15.00		18.00	^48^
**Infection to death**						
*Rivers et al.* ^48^ *(mean)*			13.31		10.38	^48^
**Case Fatality Rate (estimate, (95% CI), n)**						
*WHO ERT 2014 (based on current status)*	37.7 (36.1, 39.2), *n*=3747	57.5 (53.7, 61.1), *n*=677	34.7 (32.4, 37.1), *n*=1616	40.0 (19.8–64.3), *n*=15	31.6 (29.3, 34.1), *n*=1439	^16^
*Althaus* ^36^ *(based on reported deaths)*		74 (72, 75), *n*=607	71 (69, 74), *n*=1082		48 (47, 50), *n*=910	^36^
**All cases (based on definitive outcome)**						
*WHO ERT 2014*	70.8 (68.6, 72.8), *n*=1737	70.7 (66.7, 74.3), *n*=542	72.3 (68.9, 75.4), *n*=739	45.5 (21.3, 72), *n*=11	69.0 (64.5, 73.1), *n*=445	^16^
*WHO ERT 2015*	70.4 (69.2, 71.6), *n*=5616					^17^
*Althaus et al.* ^61^				39 (14, 71), *n*=20		^61^
*Fasina et al.* ^54^				40 (22, 61), *n*=20		^54^
**All hospitalized cases**						
*WHO ERT 2014 (based on definitive outcome)*	63.5 (60.5, 66.3), *n*=1100	63.4 (58.6, 67.9), *n*=434	66.6 (61.3, 71.6), *n*=338	100 (2.5, 100), *n*=1	61 (55.4, 66.4), *n*=318	^16^
*WHO ERT 2015 (based on definitive outcome)*	60.7 (59.2, 62.3), *n*=3839					^17^
*Rivers et al.* ^48^			0.500		0.750	^48^
**Subset of Cases**						
*Faye et al. 2014 (Conakry)*		54 (49, 63), *n*=152				^41^
R_t_ refers to effective reproduction number.						
μ=mean; Med=median; s.d.=standard deviation.						
WHO ERT is the World Health Organization Ebola Response Team^16,17^.						

*here all countries refer to Guinea, Liberia and Sierra Leone.

^†^R_*0*_ of the index case arriving by air travel from Liberia; R_t_ estimated to be below 1 within 15 days (95% CI 11–21 days) after arrival of the index case from Liberia^36^

^‡^Authors refer to generation time, but have estimated the serial interval and assumed serial interval is the same as generation time.

**Table 7:** Parameter Estimates for Marburg

**Parameter**	**Estimate**
**Generation Time Distribution (days) (mean, 95% CI, s.d., n)** * ^55^	9 (8.2, 10), 5.4, *n*=374
***R***_***0***_ **(estimate, 95% CI)**	1.59 (1.53, 1.66)
**Delay Distributions (days)**	
Symptom onset to death (range of medians)	7–8
*Ajelli & Merler (median, range, n)* ^55^	Med=7 (5–9), *n*=329
*Colebunders et al. (median, range, n)* ^57^	Med=8 (2–16), *n*=40
**Case Fatality Rate** † **(range of estimates)**	0.78–0.88
*Ajelli & Merler (estimate, 95% CI, n, year of outbreak)* ^55^	0.88 (0.84–0.91), *n*=374, 2005
*Bausch et al. (estimate, n, year of outbreak)* ^56^	0.83, *n*=(NP), 1998
*Colebunders et al. (estimate, n, year of outbreak)* ^57^	0.78, *n*=77, 1998
Med=median; s.d.=standard deviation; (NP)=Not Provided.	

*Authors refer to generation time, but have estimated the serial interval and assumed serial interval is the same as generation time.

^†^See Fig. 2 for CFR estimates.

**Table 8:** Risk factors for human-to-human transmission for infection with EVD

**Risk Factors for infection**	**OR, PPR, or RR**	**95% CI**	**Virus**	**References**
**Physical Contact**				
Sharing bed with a patient during their late stage of illness	RR=2.2	1.2, 4.2	Zaire	^39^
Slept in the same hut on the same mat	PPR=2.78	1.15, 6.70	Sudan	^45^
Touch cadaver	RR=2.1	1.1, 4.2	Zaire	^39^
Touch body of deceased person	PPR=1.95	0.91, 4.17	Sudan	^45^
Contact with known suspected case	OR=2.7	1.35, 5.24	Bundibugyo	^47^
Contact with confirmed case	RR=3.21	1.53, 6.75	Zaire	^71^
Intimate contact: nursing care	OR=5.1	1.31, 15.48	Sudan	^24^
Receiving injections	OR=7.4	1.6, 33.2	Marburg	^56^
Caring for patients at early stage of illness	PPR=6.0	1.32, 27.10	Sudan	^45^
Cared for the patient until the patient’s death at hospital	PPR=8.57	1.95, 37.66	Sudan	^45^
Cared for the patient until the patient’s death at home	PPR=13.33	3.20, 55.59	Sudan	^45^
Contact with body fluids	PPR=5.3	2.14, 13.14	Sudan	^45^
Direct physical contact with a sick person	PPR=3.53	0.52, 24.11	Sudan	^45^
**Non-Physical Contact**				
Age (>18 years old)	RR=3.6	1.3, 10.1	Zaire	^39^
Sharing a meal with a patient during their late stage of illness	RR=2.2	1.2, 4.0	Zaire	^39^
Conversation with a patient during their late stage of illness	RR=3.9	1.2, 12.2	Zaire	^39^
Shared meals with a sick person	PPR=1.94	0.89, 4.22	Sudan	^45^
Washed clothes of a sick person	PPR=1.68	0.78, 3.60	Sudan	^45^
Slept in the same hut	PPR=2.16	0.90, 5.19	Sudan	^45^
Profession- Miner	OR=13.9	3.1, 62.1	Marburg	^56^
Travelled before illness	OR=2.1	1.0, 4.5	Bundibugyo	^47^
Hospitalized or visited hospital	OR=2.6	1.4, 4.9	Bundibugyo	^47^
Consulted traditional healer	OR=0.2	0.01, 1.5	Bundibugyo	^47^
**Funeral-associated contact**				
Ritual hand washing during funeral	PPR=2.25	1.08, 4.72	Sudan	^45^
Communal meal during funeral	PPR=2.84	1.35, 5.98	Sudan	^45^
Funeral rites participation	OR=4.22	2.2, 8.2	Bundibugyo	^47^
PPR=Prevalence Proportion Ratio; RR=Risk Ratio; OR=Odds Ratio.				
